# Mapping Metastatic Spread in Uterine Sarcoma: A Population-Based Analysis of First Metastatic Patterns and Outcomes

**DOI:** 10.3390/cancers18091415

**Published:** 2026-04-29

**Authors:** Paolo Gennari, Atanas Ignatov

**Affiliations:** Department of Obstetrics and Gynecology, Otto-Von-Guericke University, Gerhard-Hauptmann-Str. 35, 39108 Magdeburg, Germany

**Keywords:** uterine sarcoma, distant metastases, metastasis pattern, survival analysis, population-based registry, leiomyosarcoma, endometrial stromal sarcoma, prognostic factors

## Abstract

**Objective:** To characterize the frequency, timing, patterns of first distant metastasis, and post-metastatic survival in uterine sarcoma using population-based registry data. **Methods:** This study included all patients diagnosed with uterine sarcoma between 2000 and October 2025 in the regional cancer registry of Saxony-Anhalt, Germany. Patients with carcinosarcoma were excluded. Metastatic disease was classified as primary (present at diagnosis or ≤3 months) or metachronous (>3 months). Metastatic patterns were analyzed based on the first metastatic presentation only. Overall survival (OS), recurrence-free survival (RFS), and post-metastatic OS were estimated using Kaplan–Meier methods. RFS was defined as the interval from confirmed tumor-free status after primary therapy to first recurrence or death, and was restricted to patients who achieved tumor-free status (n = 114). Multivariable Cox regression analyses for OS and RFS were performed with administrative censoring at 5 years. **Results:** A total of 155 patients with uterine sarcoma were included. During follow-up, 54 patients (34.8%) were diagnosed with metastatic disease, of whom 30 (55.6%) presented with primary metastatic disease. Lung was the most frequent site of first metastasis, followed by bone, peritoneum, and liver; 43.4% of metastatic patients had multiple synchronous metastatic sites at first presentation. Median time to first metastasis was short, with several metastatic sites showing median values of zero months, reflecting the high proportion of primary metastatic disease. Median post-metastatic OS was 12 months. Advanced FIGO (Fédération Internationale de Gynécologie et d’Obstétrique) stage and failure to achieve tumor-free status after primary therapy were independently associated with worse OS, whereas histologic subtype was not. **Conclusions:** In this population-based cohort, metastatic disease occurred in more than one-third of patients with uterine sarcoma and was frequently present at diagnosis. Lung metastases predominated as the first site of distant spread, and post-metastatic survival was poor. These findings underscore the importance of comprehensive staging at diagnosis and highlight the aggressive metastatic behavior of uterine sarcoma in real-world practice.

## Simple Summary

Uterine sarcomas are rare but aggressive cancers that often spread early to distant organs. However, detailed information on how and when these metastases occur, and how they affect survival, is still limited. In this study, we analyzed data from a large regional cancer registry to better understand the patterns of metastatic spread in patients with uterine sarcoma. We found that more than one-third of patients develop metastatic disease, often already present at diagnosis. The lungs were the most common site of first metastasis, and patients with multiple metastases had worse outcomes. Survival after metastasis was generally poor. These findings provide important real-world insights into how uterine sarcoma progresses and may help improve diagnostic strategies, patient counseling, and future treatment approaches.

## 1. Introduction

Uterine cancers represent the sixth most common cancer in women worldwide, with 420,368 new cases diagnosed globally in 2022, according to GLOBOCAN estimates [1]. Among these, uterine sarcomas are rare but highly aggressive malignancies, accounting for approximately 3–7% of all uterine cancers and associated with substantially poorer outcomes compared with endometrial carcinoma [2,3,4]. These tumors are characterized by early hematogenous dissemination, a high risk of recurrence, and marked clinical heterogeneity across histologic subtypes [3,5,6]. Leiomyosarcoma, endometrial stromal sarcoma, and adenosarcoma represent the most common subtypes, each exhibiting distinct patterns of disease progression and prognosis [3]. The annual incidence of uterine sarcoma is less than 2 per 100,000 women, with a median age at diagnosis of approximately 56 years [6]. Despite their rarity, these tumours carry a disproportionate mortality burden: five-year overall survival rates for leiomyosarcoma, endometrial stromal sarcoma, and adenosarcoma have been reported at 65.3%, 78.3%, and 89.5%, respectively, in large multicentre series [7]. The growing global burden of uterine cancer—driven by an aging population, rising obesity rates, and increasing incidence in low- and middle-income countries—further underscores the need for improved understanding of disease biology and metastatic behaviour [1,8].

Prognostic assessment in uterine sarcoma has traditionally relied on clinical and pathologic variables such as FIGO stage and extent of disease at diagnosis. Population-based and institutional studies consistently demonstrate that advanced stage at presentation is independently associated with inferior survival [2,3,5,6]. Prognostic staging in uterine sarcoma relies on the 2009 FIGO classification, which introduced dedicated staging systems for leiomyosarcoma and endometrial stromal sarcoma (combined) and a separate system for adenosarcoma, reflecting their distinct biological behaviour and patterns of spread [9,10]. In adenosarcoma, the presence of sarcomatous overgrowth—defined as pure high-grade sarcoma occupying at least 25% of the tumour—represents the key determinant of aggressive behaviour and is associated with substantially worse prognosis [10,11]. However, existing prognostic models largely focus on overall outcomes and do not systematically incorporate detailed information on the timing, distribution, and pattern of distant metastatic spread, despite the potential relevance of these factors for patient counseling, surveillance strategies, and clinical decision-making.

Large-scale datasets, including analyses derived from the National Cancer Database (NCDB), have provided valuable insights into survival outcomes across uterine sarcoma subtypes [12]. Nevertheless, these datasets often lack granular, organ-specific information on first metastatic presentation, as well as precise characterization of synchronous versus solitary metastatic disease. Conversely, single-institution series may offer more detailed clinical data but are inherently limited by small sample sizes and referral bias, restricting their generalizability [13].

Against this background, population-based cancer registries represent a critical resource for characterizing metastatic behavior in rare malignancies such as uterine sarcoma. In the present study, we analyzed a comprehensive cohort of patients with uterine sarcoma derived from the regional cancer registry of Saxony-Anhalt, Germany. The aim of this study was to characterize the frequency, timing, patterns of first distant metastatic spread, and post-metastatic survival outcomes in a population-based cohort of patients with uterine sarcoma, with the goal of providing real-world data to inform risk-adapted surveillance and clinical management strategies.

## 2. Materials and Methods

### 2.1. Study Design and Participants

**Figure 1:**
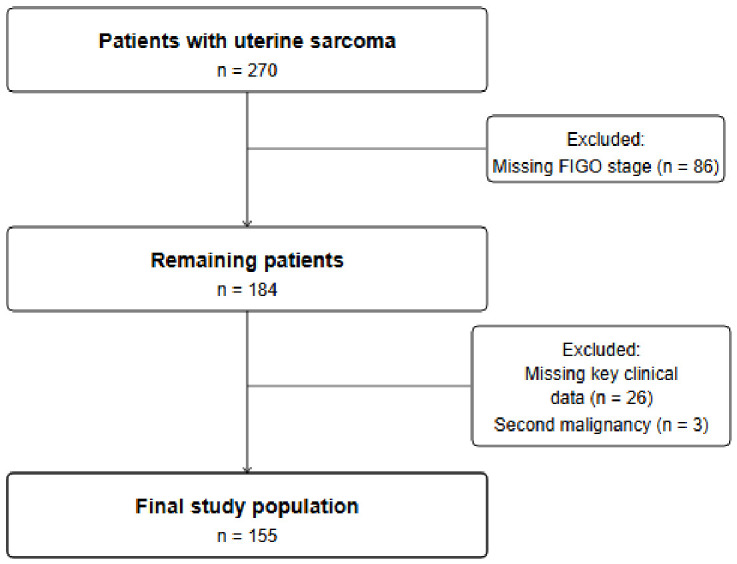
**Patient selection flowchart.** Flow diagram illustrating patient identification, exclusions, and the final study cohort.

This study included all patients diagnosed with uterine sarcoma between 1 January 2000 and 31 October 2025, as recorded in the regional cancer registry of Saxony-Anhalt, Germany. Case identification was based on histologically confirmed diagnoses documented in the registry. Patients were included irrespective of initial treatment strategy or therapeutic intent. Vital status was continuously updated through linkage with official population registries, ensuring a high level of completeness for mortality data. Patients were censored on 31 October 2025, which was the last date of available follow-up. Additional exclusions were applied after data validation for miscoded diagnoses and missing key staging or outcome data required for survival and metastatic pattern analyses. The patient selection process is detailed in Figure 1.

After application of all exclusion criteria, the final study population comprised 155 patients. As this study is based on a population-based cancer registry with a defined catchment area (Saxony-Anhalt, Germany) and time period (2000–2025), the sample size was determined by the available registry data and was not based on a pre-specified power calculation. The final cohort of 155 patients represents one of the larger single-institution or regional registry series on uterine sarcoma reported to date, providing adequate statistical power for the primary descriptive and survival analyses. However, the relatively small absolute numbers in histologic subgroups preclude formal hypothesis testing by subtype, and all subgroup analyses should be considered exploratory. Clinical, pathological, treatment-related, and follow-up variables were extracted directly from the registry database.

### 2.2. Data Collection

Clinical, pathological, and treatment-related data were obtained from the population-based cancer registry of Saxony-Anhalt, Germany, which collects standardized, mandatory cancer notifications from hospitals and outpatient providers across the region. Recorded variables included age at diagnosis, histologic subtype according to ICD-O coding, FIGO stage at diagnosis, ECOG performance status, primary treatment modalities, surgical resection status, and survival outcomes.

Information on disease recurrence and distant metastases was extracted from follow-up and progression reports submitted to the registry. Dates of first recurrence, first distant metastasis, and death were recorded when available. Information on vital status and date of death was supplemented by linkage with the regional population registry (Melderegister) to ensure completeness of mortality data. For patients who achieved tumor-free status after primary therapy, the date of confirmed tumor-free status was used as the reference point for recurrence-free survival (RFS), defined as the interval from this date to first recurrence (local or distant) or death.

Histologic subtypes were grouped into leiomyosarcoma (including epithelioid variants), endometrial stromal sarcoma (low- and high-grade combined), adenosarcoma (including cases with sarcomatous overgrowth), and other rare uterine sarcomas. FIGO stage was recorded according to contemporary staging criteria and additionally categorized into a binary variable (FIGO I–II vs. III–IV) for survival analyses. It is noted that the 2009 FIGO staging system introduced separate staging classifications for leiomyosarcoma/endometrial stromal sarcoma and adenosarcoma [9,10]. In the present study, FIGO stage was applied as recorded in the population-based registry at the time of diagnosis, consistent with real-world clinical documentation and registry-based methodology. Systematic verification of subtype-specific staging consistency across the entire study period was not feasible within the registry design.

Primary metastatic disease was defined as the presence of distant metastases at diagnosis or within three months thereafter; metastases diagnosed beyond this interval were classified as metachronous. For metastatic pattern analyses, only the first metastatic presentation was considered. Organ-specific metastatic involvement at first presentation was recorded for the lungs, liver, bone, lymph nodes, peritoneum, brain, and other sites. In cases of synchronous multi-organ involvement at first metastatic presentation, metastases were classified as multiple.

### 2.3. Definitions

Distant metastasis was defined as radiologically or histologically confirmed metastatic disease at sites distant from the primary uterine tumor, including both hematogenous and peritoneal metastatic spread. Metastatic disease present at diagnosis or diagnosed within three months thereafter was classified as primary metastatic disease, whereas metastases occurring more than three months after diagnosis were classified as metachronous.

For time-to-metastasis analyses, metastatic events diagnosed within three months of diagnosis were considered synchronous with the primary tumor. For graphical presentation purposes only, these events were assigned a time to metastasis of zero months, reflecting primary metastatic presentation.

At first metastatic presentation, involvement of a single distant organ site was classified as a single first metastasis, whereas simultaneous involvement of two or more distant organ sites was classified as multiple synchronous first metastases.

Tumor-free status after primary therapy was defined as the absence of clinical or radiological evidence of disease following completion of initial treatment. The date of confirmed tumor-free status was used as the reference point for recurrence-free survival (RFS) analyses.

Recurrence-free survival (RFS) was defined as the interval from the date of confirmed tumor-free status after primary therapy to the date of first recurrence (local or distant) or death from any cause. RFS analyses were restricted to patients who achieved tumor-free status after primary therapy (n = 114), as patients with persistent or primary metastatic disease cannot be considered to have a recurrence-free interval. This definition differs from the classical Disease-Free Survival (DFS) endpoint, which is typically measured from the date of diagnosis or surgery and includes all patients regardless of residual disease status. The use of tumor-free status as the reference point was chosen to provide a clinically meaningful measure of recurrence risk in patients who were initially rendered free of disease, and is consistent with RFS definitions used in the surgical oncology literature. Patients without recurrence or death were censored at the date of last follow-up.

Overall survival (OS) was defined as the interval from date of initial diagnosis to death from any cause or last follow-up. Post-metastatic overall survival was defined as the interval from date of first documented distant metastasis to death or last follow-up. Rare metastatic sites, including pleural, splenic, cutaneous, and other uncommon localizations, were grouped as ‘other’ for descriptive analyses.

### 2.4. Statistical Analysis

Survival distributions were estimated using the Kaplan–Meier method and compared between groups using the log-rank test, based on the full available follow-up. Cox proportional hazards regression was used to estimate hazard ratios (HRs) with 95% confidence intervals (CI) for OS and RFS. For Cox regression analyses, follow-up time was administratively censored at 60 months (5 years) to ensure model stability and comparability across subgroups. In univariable Cox models for OS, FIGO stage was modeled as an ordinal variable (per-stage increase from I to IV), whereas in multivariable OS models FIGO stage was dichotomized (I–II vs. III–IV) to reduce overparameterization and improve model stability. For RFS analyses, FIGO stage was modeled as a binary variable (I–II vs. III–IV) in both univariable and multivariable Cox models due to the reduced sample size after exclusion of patients who did not achieve tumor-free status.

Predefined clinically relevant variables were entered into univariable and multivariable Cox models. For OS, multivariable analyses included FIGO stage, primary metastatic disease, and tumor-free status after primary therapy; age at diagnosis was included in sensitivity analyses. For RFS, multivariable analyses included FIGO stage and histologic group.

Because uterine sarcomas are frequently diagnosed postoperatively, complete staging is often performed after histologic confirmation. Therefore, metastatic disease documented within three months of the initial diagnosis was classified as metastatic disease at diagnosis (primary metastatic disease), assuming that metastatic spread was already present but detected during delayed staging. For descriptive time-to-metastasis analyses, the observed interval in months from diagnosis to first documented metastasis was retained.

Metastatic patterns were analyzed descriptively, including the frequency of distant metastases, timing of metastatic disease (primary vs. metachronous), distribution of first metastatic sites, single versus multiple synchronous first metastases, and organ-specific time to first metastasis. Organ-based subgroup analyses were performed for descriptive purposes without formal hypothesis testing.

Categorical variables were compared using chi-square or Fisher’s exact tests where appropriate. Statistical significance was defined as a two-sided *p* value < 0.05. All analyses were performed using IBM SPSS Statistics version 29 (IBM Corp., Armonk, NY, USA). Given the observational, registry-based design of this study, no formal a priori sample size calculation was performed. The adequacy of the sample for the primary analyses was assessed post hoc: the 5-year OS Cox regression model included three covariates with 55 events, yielding an events-per-variable ratio of approximately 18, which is considered sufficient for stable model estimation. Analyses stratified by histologic subtype or tumor grade were performed as exploratory analyses only, in view of the limited number of patients within individual subgroups. The selection of statistical tests was guided by the nature and distribution of the data. The Kaplan–Meier method was selected as the standard non-parametric estimator for survival distributions in the presence of censored observations, requiring no distributional assumptions. Groups were compared using the log-rank (Mantel–Cox) test, which is appropriate when the proportional hazards assumption is approximately met, and survival curves do not substantially cross. Cox proportional hazards regression was employed to estimate adjusted hazard ratios with 95% confidence intervals, allowing simultaneous control for multiple prognostic covariates; administrative censoring at 60 months was applied to ensure model stability and a sufficient events-per-variable ratio. For comparisons of categorical variables, Pearson chi-square tests were used when all expected cell frequencies were at least five; Fisher’s exact test was applied when expected cell frequencies fell below this threshold, providing exact *p*-values without relying on large-sample approximations. The Mann–Whitney U test was used for comparisons of continuous variables between two independent groups when normality could not be assumed, as is standard for registry-based cohort data.

## 3. Results

### 3.1. Study Population

Between January 2000 and 31 October 2025, patients with a registry-based diagnosis of uterine sarcoma were identified from the population-based cancer registry of Saxony-Anhalt, Germany. After exclusion of miscoded carcinosarcomas, secondary malignancies, and cases with incomplete staging or follow-up information, 155 patients were included in the final analysis.

### 3.2. Baseline Characteristics

Baseline characteristics are summarized in Table 1.

Median age at diagnosis was 59 years (IQR 51–72). Leiomyosarcoma was the most common histologic subtype (43.2%), followed by endometrial stromal sarcoma (36.8%), adenosarcoma (10.3%), and other rare sarcomas (9.7%). At diagnosis, 65.2% of patients had FIGO stage I disease, 7.1% stage II, 8.4% stage III, and 19.4% stage IV. Overall, 54 of 155 patients (34.8%) were diagnosed with metastatic disease, including 30 patients (19.4% of the total cohort) with primary metastatic disease. Tumor-free status after primary therapy was achieved in 114 of 150 assessable patients (76.0%). Median follow-up was 53 months (IQR 17–117 months).

### 3.3. Tumor Grade Analysis

**Figure 2:**
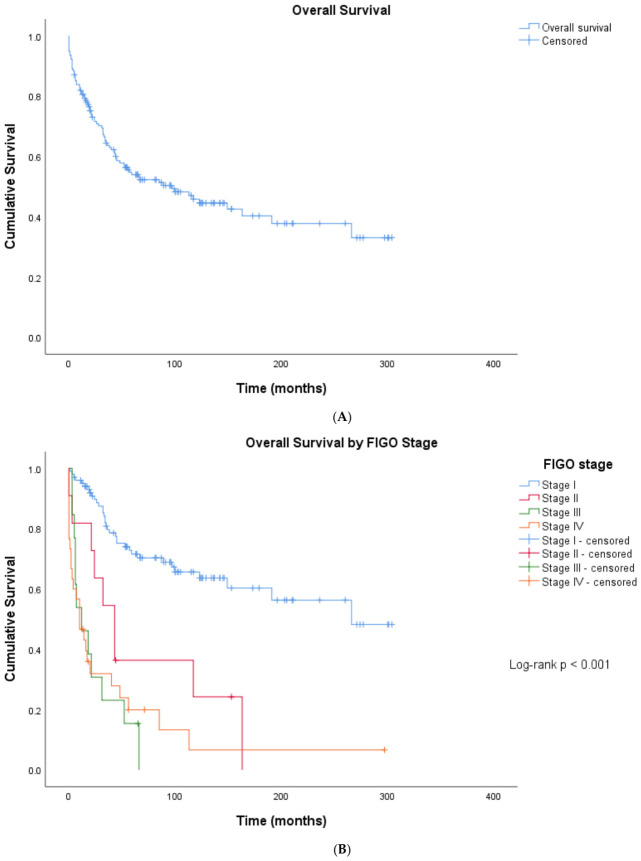

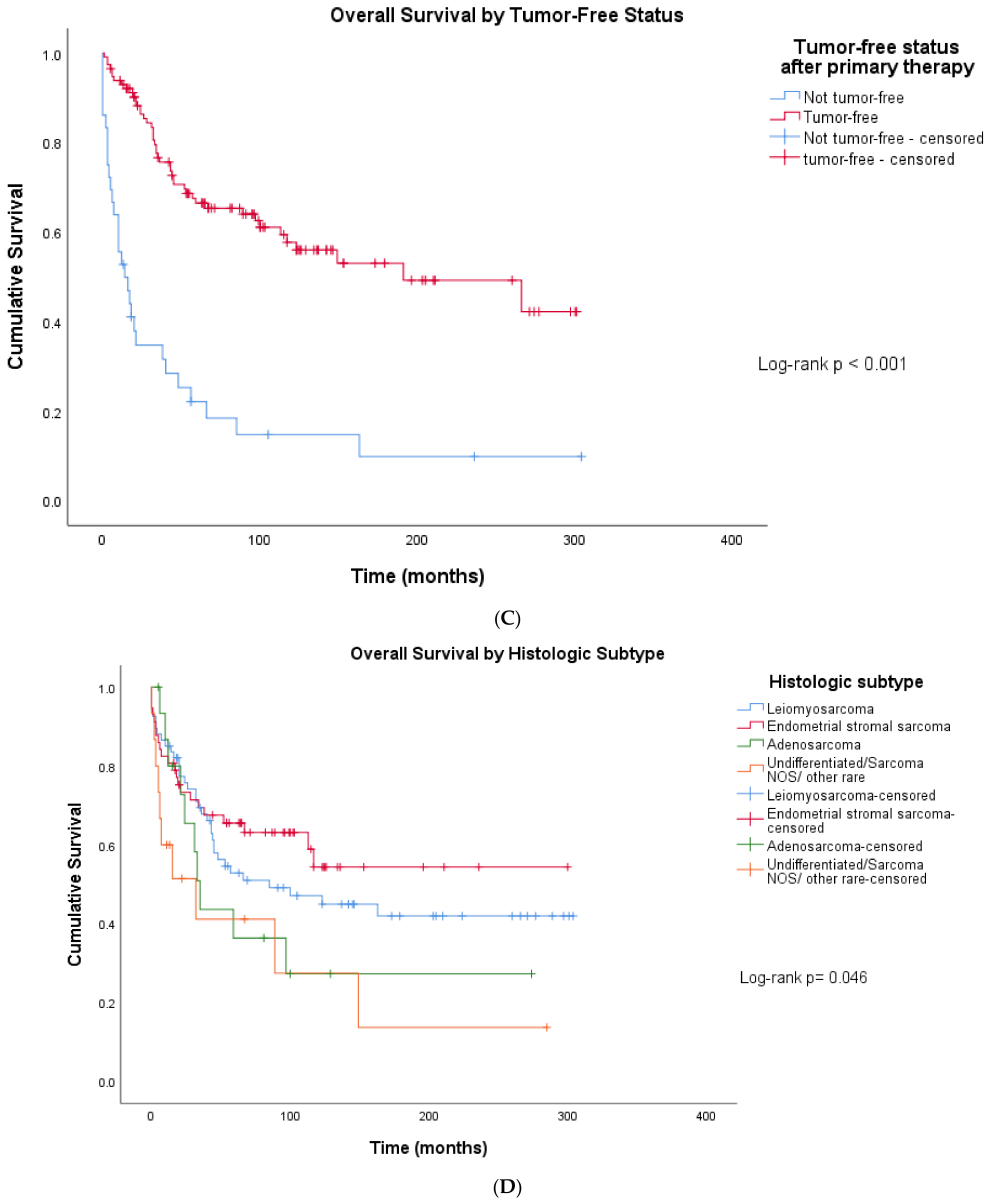

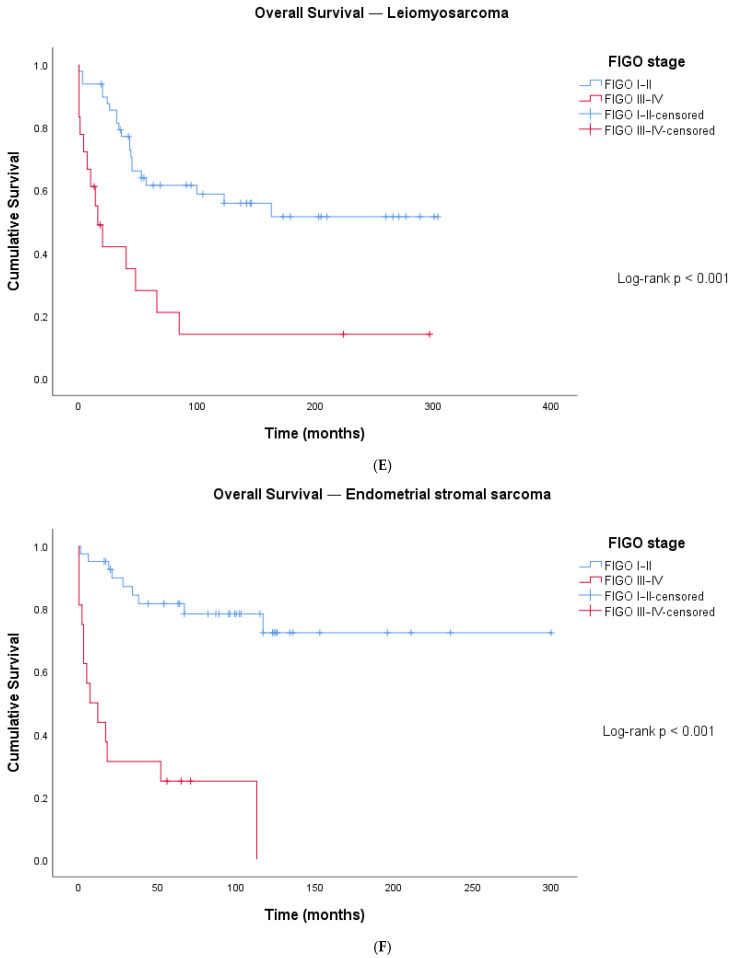

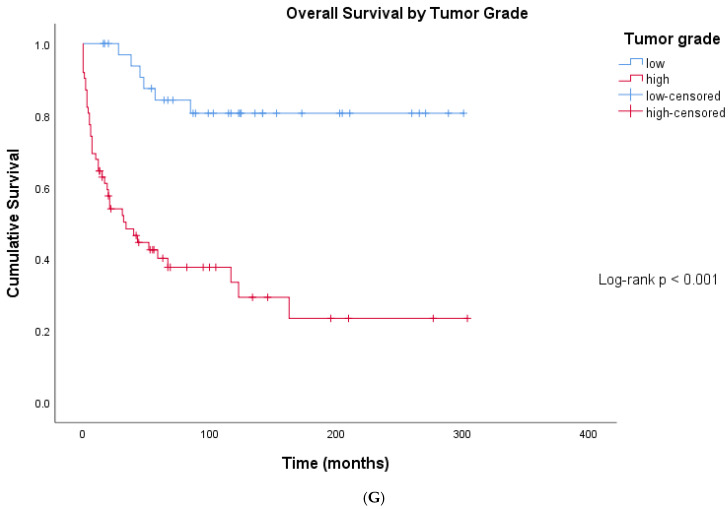
(**A**) Overall survival of the entire study cohort (n = 155). Kaplan–Meier estimate. OS, overall survival. (**B**) Overall survival stratified by FIGO stage (n = 155). Kaplan–Meier estimates. Log-rank *p* < 0.001. FIGO, Fédération Internationale de Gynécologie et d’Obstétrique. (**C**) Overall survival stratified by tumour-free status after primary therapy (n = 150 assessable patients). Kaplan–Meier estimates. Log-rank *p* < 0.001. Tick marks indicate censored observations. (**D**) Overall survival stratified by histologic subtype (n = 155). Kaplan–Meier estimates. Log-rank *p* = 0.046. Tick marks indicate censored observations. LMS, leiomyosarcoma; ESS, endometrial stromal sarcoma. (**A**–**D**) Overall survival (OS) analyses. (**E**) Overall survival stratified by FIGO stage in leiomyosarcoma (n = 67). Kaplan–Meier estimates. Log-rank *p* < 0.001. FIGO, Fédération Internationale de Gynécologie et d’Obstétrique. Tick marks indicate censored observations. (**F**) Overall survival stratified by FIGO stage in endometrial stromal sarcoma (n = 57). Kaplan–Meier estimates. Log-rank *p* < 0.001. Tick marks indicate censored observations. (**E**,**F**) KM OS stratified by FIGO within LMS and ESS. (**G**) Overall survival stratified by tumour grade (n = 97 with available grade data). Kaplan–Meier estimates. This is an exploratory analysis restricted to patients with known tumour grade. Log-rank *p* < 0.001. Tick marks indicate censored observations.

Tumor grade data were available for 97 of 155 patients (62.6%); grade was missing in 58 patients (37.4%), with missing grade significantly associated with histologic subtype (*p* = 0.005), particularly adenosarcoma (68.8% missing). Sensitivity analyses demonstrated that patients with missing grade data did not differ significantly from those with known grade with respect to FIGO stage (*p* = 0.479), metastatic status (*p* = 0.532), overall survival (*p* = 0.290), or age at diagnosis (*p* = 0.631). Among the 97 patients with known grade, 35 (36.1%) had low-grade, and 62 (63.9%) had high-grade tumours. The metastatic rate differed markedly by grade: 11.4% in low-grade versus 45.2% in high-grade tumours (*p* = 0.001). High-grade histology was significantly associated with advanced FIGO stage (*p* = 0.004). Kaplan–Meier analysis demonstrated a significant survival difference by grade (log-rank *p* < 0.001): low-grade tumours showed excellent survival (6 deaths [17.1%]; median OS not reached), while high-grade tumours showed poor prognosis (39 deaths [62.9%]; median OS 34 months, 95% CI 11–57; Figure 2G). In exploratory multivariable Cox regression restricted to 95 patients with complete covariate data, high-grade histology was independently associated with worse 5-year OS (HR 4.84, 95% CI 1.85–12.63, *p* = 0.001), alongside FIGO stage III–IV (HR 4.15, 95% CI 1.79–9.62, *p* = 0.001). These grade-based analyses should be interpreted as exploratory, given the 37.4% missing grade data (Supplementary Tables S3 and S4).

### 3.4. Overall Survival

Kaplan–Meier estimates for overall survival (OS) are shown in Figure 2.

OS decreased with advancing FIGO stage. Survival was longer in patients who achieved tumor-free status after primary therapy compared with those with residual disease. Patients with primary metastatic disease had shorter OS than patients without metastases at diagnosis.

### 3.5. Recurrence-Free Survival

Recurrence-free survival (RFS) analyses were restricted to patients who achieved tumor-free status after primary therapy (n = 114). RFS was defined as the interval from confirmed tumor-free status to first recurrence (local or distant) or death (see Definitions). Kaplan–Meier curves are shown in Supplementary Figure S1. A trend toward shorter RFS was observed in patients with advanced FIGO stage (III–IV vs. I–II), although this did not reach statistical significance (log-rank *p* = 0.065). Stratification by histologic subtype showed largely overlapping survival curves. Multivariable analyses of RFS are presented in Supplementary Table S1.

### 3.6. Multivariable Survival Analyses

Results of multivariable Cox regression analyses with administrative censoring at 60 months are presented in Table 2.

For 5-year OS, FIGO stage III–IV was independently associated with worse survival (hazard ratio [HR] 3.43, 95% confidence interval [CI] 1.60–7.35, *p* = 0.002), while tumor-free status after primary therapy was independently associated with improved OS (HR 0.42, 95% CI 0.21–0.85, *p* = 0.016). Primary metastatic disease was not independently associated with OS after adjustment. In the multivariable analysis for 5-year RFS, neither FIGO stage grouping nor histologic subtype was independently associated with RFS. In an exploratory sensitivity analysis restricted to tumor-free patients, leiomyosarcoma histology was associated with a numerically shorter RFS compared with other uterine sarcomas, reaching statistical significance after adjustment for FIGO stage (Supplementary Table S2). Given the exploratory and post-hoc nature of this analysis, these findings should be interpreted with caution.

### 3.7. Subgroup Analyses by Histologic Subtype and FIGO Stage

Kaplan–Meier OS analyses stratified by histologic subtype demonstrated significant differences in survival (log-rank *p* = 0.046). Endometrial stromal sarcoma showed the most favourable prognosis (median OS not reached, 38.6% mortality), while leiomyosarcoma showed intermediate survival (median OS 85 months, 95% CI 2–168, 52.2% mortality). Adenosarcoma and other/undifferentiated sarcomas showed the poorest outcomes (median OS 35 months [95% CI 28–42] and 32 months [95% CI 0–69] respectively), although these subgroups were small (n = 16 and n = 15) and results should be interpreted as exploratory.

Within the two largest histologic subgroups, FIGO stage was a major determinant of OS. In leiomyosarcoma (n = 67), median OS was not reached in FIGO I–II versus 16 months (95% CI 5–27) in FIGO III–IV (log-rank *p* < 0.001). In endometrial stromal sarcoma (n = 57), median OS was not reached in FIGO I–II versus 7 months (95% CI 0–21) in FIGO III–IV, with 81.3% mortality in the advanced-stage group (overall log-rank *p* < 0.001, adjusted for histologic subtype; Figure 2E,F). The metastatic rate also differed significantly by histologic subtype (*p* = 0.018), with leiomyosarcoma showing the highest rate (47.8%) and adenosarcoma the lowest (12.5%).

### 3.8. Metastasis Pattern Analysis

Among patients with metastatic disease, 30 (55.6%) had primary metastatic disease and 24 (44.4%) developed metachronous metastases (>3 months after diagnosis). Among metachronous cases, the median time to first metastasis was 23.5 months (IQR 9.5–37.8 months; range 4–71 months). The metastatic site distribution in the metachronous subgroup closely mirrored that of the overall cohort, with lung as the predominant first metastatic site (66.7%), followed by bone (29.2%) and peritoneum (16.7%). Post-metastatic survival did not differ significantly between patients with metachronous and primary metastatic disease (median 12 vs. 14 months, log-rank *p* = 0.815), underscoring the poor prognosis once metastatic disease has developed, regardless of its timing (Figure 4C).

#### Pattern of First Metastatic Presentation

At first metastatic presentation, 32 patients (59.3%) had a single metastatic site, whereas 22 patients (40.7%) presented with multiple synchronous metastatic sites. The distribution of first distant metastatic sites is shown in Table 3.

Lung metastases were most frequent (68.5%), followed by bone (25.9%), peritoneum (18.5%), liver (16.7%), other sites (22.2%), lymph nodes (9.3%) and brain (3.7%). Percentages exceed 100% due to synchronous multi-organ involvement.

### 3.9. Time to First Metastasis

Among patients with metastatic disease, median time to first metastasis was 0.5 months (IQR 0–17.5 months) (Table 3). This finding reflects the high proportion of primary metastatic disease, as synchronous metastases were assigned a time to metastasis of zero months for descriptive purposes. Median time to first metastasis was 0 months for lung, liver, brain, and other sites. Bone metastases occurred later, with a median time of 3 months (IQR 0–36 months), while lymph node metastases had a median time of 6 months (IQR 0–25 months). Peritoneal metastases occurred early, with a median time of 0.5 months (IQR 0–8 months).

### 3.10. Post-Metastatic Overall Survival

**Figure 3:**
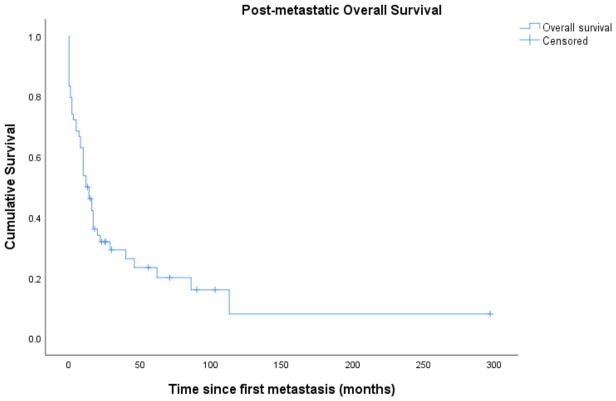
Post-metastatic overall survival. Kaplan–Meier curve depicting overall survival calculated from the date of first distant metastasis among patients who developed metastatic disease.

Post-metastatic overall survival is shown in Figure 3.

**Figure 4:**
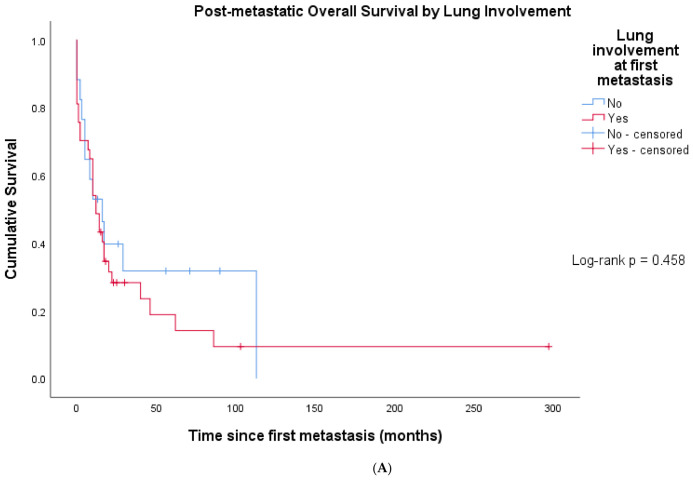

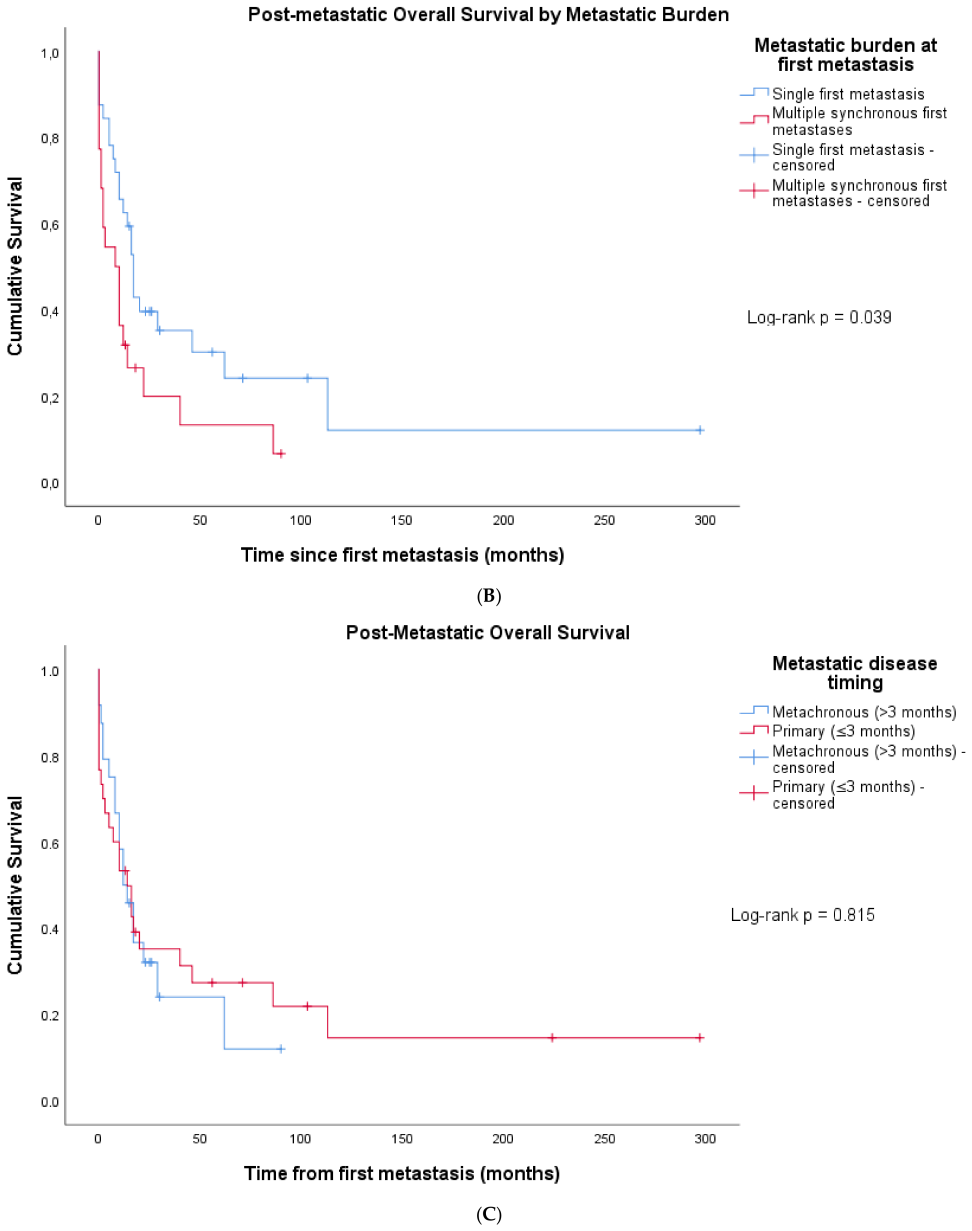
Post-metastatic overall survival stratified by metastatic characteristics. (**A**) Post-metastatic overall survival stratified by lung involvement. Kaplan–Meier curves comparing overall survival from the date of first distant metastasis between patients with versus without lung involvement at first metastatic presentation. Log-rank *p* = 0.458. (**B**) Post-metastatic overall survival stratified by metastatic burden. Kaplan–Meier curves comparing overall survival from the date of first distant metastasis between patients with single versus multiple synchronous first distant metastases. Log-rank *p* = 0.039. (**C**) Post-metastatic overall survival by timing of metastatic disease (n = 54). Kaplan–Meier estimates comparing patients with metachronous metastases (>3 months from diagnosis, n = 24) vs. primary metastatic disease (≤3 months, n = 30). Log-rank *p* = 0.815. Tick marks indicate censored observations.

Median post-metastatic OS was 12.0 months (95% CI 6.9–17.1 months). Median post-metastatic OS was 12 months in patients with lung involvement at first metastasis and 16 months in patients without lung involvement (log-rank *p* = 0.458). (Figure 4A). Patients with multiple synchronous first metastases had significantly shorter post-metastatic OS compared with those with a single metastatic site (median 8 months vs. 17 months, respectively; log-rank *p* = 0.039) (Figure 4B).

## 4. Discussion

Uterine sarcomas are rare and biologically aggressive malignancies, for which population-based data on metastatic dissemination and post-metastatic outcomes remain limited. In this population-based cohort, we analyzed metastatic frequency, timing, first metastatic patterns, and post-metastatic survival across all uterine sarcoma subtypes using long-term registry follow-up and clearly defined metastatic endpoints. Our findings demonstrate a high burden of metastatic disease, frequent primary metastatic presentation, and poor survival after distant metastasis.

Approximately one-third of patients developed metastatic disease, with more than half presenting with primary metastatic disease. These rates are consistent with prior population-based analyses reporting metastatic frequencies of 25–40% in uterine sarcoma, particularly leiomyosarcoma-dominant cohorts [14,15]. The high proportion of primary metastatic disease observed in our study likely reflects the population-based design, which captures patients regardless of surgical eligibility and minimizes selection bias typical of institutional series [16]. The overall metastatic rate of 34.8% warrants contextualisation within the broader literature. Notably, restricting the metastatic pattern analysis to metachronous cases did not substantially alter the observed distribution, with lung remaining the predominant first metastatic site (66.7%), suggesting that the overall metastatic pattern is robust regardless of the timing of disease progression. The metastatic rate differed substantially across histologic subtypes: leiomyosarcoma showed the highest rate at 47.8%, followed by endometrial stromal sarcoma at 28.1%, and adenosarcoma at 12.5% (*p* = 0.018). The lower overall rate compared with some published estimates—including analyses of the SEER database reporting recurrence rates of approximately 70% for stage I uterine leiomyosarcoma [15]—can be attributed to several factors: our cohort includes all uterine sarcoma subtypes with lower metastatic potential than leiomyosarcoma; 65.2% of patients presented with FIGO stage I disease; the published SEER recurrence rate encompasses all forms of recurrence including local relapse, whereas the present study captures first distant metastasis only; and among patients with known grade, 63.9% had high-grade tumours with a metastatic rate of 45.2%, consistent with published estimates for aggressive uterine sarcomas [7].

Lung metastases were the most frequent site of first distant spread, followed by bone, peritoneum, and liver. This pattern is consistent with prior reports describing predominantly hematogenous dissemination in uterine sarcoma [17,18]. Notably, over 40% of metastatic patients presented with multiple synchronous metastatic sites at first recurrence, highlighting the systemic nature of the disease at metastatic presentation. By restricting analyses to the first metastatic event, our study provides a focused assessment of early metastatic behavior.

Time-to-metastasis analyses further support early dissemination in uterine sarcoma. Median time to first metastasis was short, with several metastatic sites demonstrating median values of zero months, reflecting metastases present at diagnosis. Bone and lymph node metastases occurred later than lung or liver metastases, consistent with previously described dissemination patterns [17,19]. These findings support current guideline recommendations for comprehensive staging at diagnosis, including thoracic imaging [16,20]. Although detailed information on imaging modalities at diagnosis was not available, the observed timing and distribution of first metastases are consistent with population-based data and current staging recommendations.

Post-metastatic survival was poor, with a median overall survival of approximately 12 months after first distant metastasis. This estimate aligns closely with prior population-based and multi-institutional studies reporting median post-metastatic survival between 12 and 18 months [14]. Patients presenting with multiple synchronous metastatic sites had significantly shorter post-metastatic survival compared with those with a single metastatic site. In contrast, lung involvement at first metastatic presentation was not associated with a statistically significant difference in post-metastatic survival. Similar trends have been reported in registry-based and NCDB analyses, including recent data on synchronous lung metastases [21].

In multivariable analyses, advanced FIGO stage and failure to achieve tumor-free status after primary therapy were independently associated with worse overall survival, whereas histologic subtype did not retain independent prognostic significance. The lack of an independent association between primary metastatic disease and overall survival in multivariable analysis is likely explained by collinearity with advanced FIGO stage, as most patients with primary metastatic disease were classified as stage IV. While uterine sarcomas are histologically heterogeneous, our population-based approach was designed to capture overall metastatic behavior across subtypes rather than subtype-specific biology. These findings are consistent with previous registry-based studies indicating that disease extent and initial disease control outweigh histologic classification in advanced-stage uterine sarcoma [12]. The absence of independent prognostic significance of histologic subtype in the multivariable model should be interpreted in light of the biological heterogeneity within each subtype group. In particular, adenosarcoma encompasses a spectrum from indolent tumours without sarcomatous overgrowth to highly aggressive tumours with sarcomatous overgrowth, which confers a prognosis comparable to high-grade leiomyosarcoma [10]. Similarly, endometrial stromal sarcoma includes both low-grade and high-grade variants with markedly different clinical behavior, defined by distinct molecular alterations such as JAZF1–SUZ12 fusions in low-grade and YWHAE–NUTM2 fusions or BCOR alterations in high-grade tumours [22,23]. The grouping of histologic subtypes for statistical analysis, necessitated by sample size constraints, may therefore obscure important within-subtype heterogeneity.

Exploratory analyses of tumor grade, available in 97 patients (62.6%), revealed a strong and biologically plausible association with both metastatic risk and survival. High-grade tumours were associated with a metastatic rate of 45.2% versus 11.4% in low-grade tumours (*p* = 0.001), and with markedly worse overall survival (median OS 34 months vs. not reached; log-rank *p* < 0.001). In exploratory multivariable analysis, high-grade histology was independently associated with worse 5-year OS (HR 4.84, 95% CI 1.85–12.63, *p* = 0.001), alongside FIGO stage. These findings are consistent with prior series demonstrating that tumor grade is among the strongest prognostic determinants in uterine sarcoma [7,24], and support the routine reporting of grade in registry databases. The absence of grade data in 37.4% of patients—primarily driven by adenosarcoma cases, for which a standardized grading system is not routinely applied—represents an important limitation and underscores the need for standardized pathologic reporting in rare uterine malignancies.

However, in an exploratory sensitivity analysis restricted to patients who achieved tumor-free status, leiomyosarcoma histology was associated with a shorter recurrence-free survival after adjustment for FIGO stage. This finding is biologically plausible given the known aggressive behavior of leiomyosarcoma, but should be interpreted cautiously due to the limited sample size and post-hoc nature of the analysis.

The findings of this study carry important implications for clinical practice and highlight several areas for future investigation. The high proportion of patients presenting with primary metastatic disease (55.6% of all metastatic cases) underscores a critical diagnostic challenge: uterine sarcomas are frequently diagnosed at an advanced stage, often because preoperative distinction from benign uterine leiomyomas remains unreliable [25,26]. To minimise the risk of inadequate staging or inadvertent tumour fragmentation at the time of surgery, several clinical strategies warrant consideration. First, a low threshold for cross-sectional imaging—including CT of the thorax, abdomen, and pelvis—should be applied in all patients with a suspicious uterine mass, particularly postmenopausal women or those with rapid tumour growth. Pelvic MRI with diffusion-weighted imaging may further stratify the risk of malignancy in suspicious uterine masses, and recent multidisciplinary consensus criteria provide a structured framework for radiologic risk assessment [27]. Second, where clinically feasible, preoperative histologic confirmation through transvaginal or transabdominal biopsy of suspicious uterine masses should be considered prior to surgical intervention. Third, patients with suspected or confirmed uterine sarcoma should be referred to centres with dedicated expertise in gynaecological oncology and soft tissue sarcoma, in line with current guideline recommendations for centralisation of rare gynaecological malignancy care [20,28]. Pooled analyses suggest that pulmonary metastasectomy may confer prolonged survival in carefully selected patients with leiomyosarcoma, with a long disease-free interval and limited disease burden as key predictors of benefit [29,30]. Future studies should prospectively evaluate the impact of metastasectomy in selected patients with oligometastatic disease, the role of morcellation on metastatic patterns, and systemic therapy outcomes in this population. The role of adjuvant systemic therapy in early-stage disease remains particularly uncertain, given that the only randomised phase III trial in uterine leiomyosarcoma (NRG Oncology/GOG) was closed prematurely for accrual futility and did not demonstrate a survival advantage for adjuvant chemotherapy [31].

The strengths of this study include its population-based design with long-term follow-up spanning 25 years, inclusion of all uterine sarcoma subtypes excluding carcinosarcoma, comprehensive mortality ascertainment through linkage with the regional population registry, and a focused assessment of first metastatic presentation with clearly defined metastatic endpoints. Several limitations merit consideration. First, as with all registry-based analyses, data quality is dependent on completeness of reporting, and the retrospective design precludes central pathology review or molecular profiling. Second, tumor grade data were available for only 97 of 155 patients (62.6%), with missing grade significantly associated with histologic subtype—particularly adenosarcoma, for which a formal grading system is not routinely applied in clinical practice. All grade-based analyses were therefore performed as exploratory analyses in patients with available data. Third, the median follow-up of 53 months, while adequate for high-grade tumours in which most recurrences occur within the first three years, may underrepresent the true long-term metastatic burden in low-grade endometrial stromal sarcoma, for which late recurrences beyond five years are well documented. Fourth, the registry-based design precluded collection of data on surgical technique, including information on uterine morcellation—a known risk factor for peritoneal dissemination in cases of undetected uterine sarcoma [32]. Fifth, no data were available on neoadjuvant or adjuvant systemic therapy, treatment of metastatic disease, including surgical management of metastases, or response to systemic treatment. Sixth, the FIGO staging system was applied as recorded in the registry at the time of diagnosis. While distinct FIGO staging classifications have existed for leiomyosarcoma/ESS and adenosarcoma since 2009 [9,10], subtype-specific staging consistency across the entire study period could not be systematically verified from registry data. Seventh, the relatively high number of cases excluded for incomplete staging or outcome data (detailed in Figure 1) reflects the long study period beginning in 2000, during which registry data completeness was lower; sensitivity analyses confirmed that excluded patients did not differ significantly from the final cohort with respect to key prognostic variables. Finally, statistical power for subgroup analyses by histologic subtype was limited due to small numbers in individual groups, particularly adenosarcoma and other rare sarcomas; all such analyses should be considered exploratory. Despite these limitations, population-based cancer registries provide a critical resource for characterising metastatic behaviour in rare malignancies such as uterine sarcoma, offering real-world data that are difficult to obtain from institutional series.

## 5. Conclusions

In conclusion, this population-based analysis demonstrates that metastatic disease occurs in more than one-third of patients with uterine sarcoma and is frequently present at diagnosis, with lung as the predominant first site of distant spread. Post-metastatic survival is poor regardless of the timing of metastatic progression, with a median post-metastatic overall survival of 12 months. Tumor grade and histologic subtype are significant determinants of metastatic risk and overall survival, with high-grade tumors showing markedly worse outcomes. Advanced FIGO stage and failure to achieve tumor-free status after primary therapy are independently associated with inferior survival. These findings underscore the importance of comprehensive staging at diagnosis, including low-threshold cross-sectional imaging, and support referral to specialised centres for suspected uterine sarcoma. Addressing the diagnostic challenge of preoperative identification of uterine sarcoma and standardising clinical pathways for suspicious uterine masses represent critical priorities for improving outcomes in this aggressive malignancy.

## Figures and Tables

**Table 1:** **Baseline characteristics of the study cohort**.

Characteristic	Total (n = 155)
Age at diagnosis, years—median (IQR)	59 (IQR 51–72)
**Histology**	
Leiomyosarcoma	67 (43.2%)
Endometrial stromal sarcoma	57 (36.8%)
Adenosarcoma	16 (10.3%)
Other sarcomas	15 (9.7%)
**FIGO stage**	
Stage I	101 (65.2%)
Stage II	11 (7.1%)
Stage III	13 (8.4%)
Stage IV	30 (19.4%)
**Metastasis (any), yes**	54 (34.8%)
**Primarily metastatic (≤3 months), yes**	30 (19.4%)
**First treatment modality**	
Surgery	146 (94.2%)
RT	2 (1.3%)
CTX	2 (1.3%)
None	5 (3.2%)
**Tumor-free after primary therapy, yes**	114/150 (76.0%)
**RT-based adjuvant therapy, yes**	28/146 (19.2%)
**Follow-up, months—median (IQR), range**	53 (IQR 17–117), 0–304

Tumor-free status after primary therapy was not assessable in 5 untreated patients. RT-based adjuvant therapy was not applicable in 9 patients (5 untreated; 4 receiving RT or CT as primary treatment).

**Table 2:** **Univariable and multivariable Cox proportional hazards regression for overall survival (OS) at 5 years**.

Variable	Univariable HR (95% CI)	*p*-Value	Multivariable HR (95% CI)	*p*-Value
FIGO stage (I–IV, per-stage increase)	1.44 (1.12–1.86)	0.005	—	—
FIGO stage (III–IV vs. I–II)	—	—	3.43 (1.60–7.35)	0.002
Primary metastasis (yes vs. no)	3.62 (2.14–6.13)	<0.001	0.65 (0.32–1.36)	0.253
Tumor-free after primary therapy (yes vs. no [reference])	0.23 (0.14–0.38)	<0.001	0.42 (0.21–0.85)	0.016

Results of multivariable Cox proportional hazards regression analyses for 5-year overall survival and recurrence-free survival. Models were administratively censored at 60 months. Hazard ratios (HRs) are reported with 95% confidence intervals (CIs). In univariable analyses, FIGO stage was modeled as an ordinal variable (per-stage increase from I to IV). In multivariable analyses, FIGO stage was entered as a binary variable (FIGO I–II vs. III–IV) to improve model stability.

**Table 3:** **Metastatic patterns and timing**.

First Metastatic Site	n (%)	Median Time to First Metastasis, Months (IQR)
Overall	54 (100)	0.5 (0–17.5)
Lung	37 (68.5)	0.0 (0–29)
Bone	14 (25.9)	3.0 (0–36)
Peritoneum	10 (18.5)	0.5 (0–8)
Liver	9 (16.7)	0.0 (0–18)
Lymph nodes	5 (9.3)	6.0 (0–25)
Brain	2 (3.7)	0.0 (0–0)
Other	11 (20.4)	0.0 (0–4)

## Data Availability

The data used in this study are derived from the Cancer Registry of Saxony-Anhalt. Due to data protection regulations, the data are not publicly available but can be obtained upon reasonable request and with permission from the registry authorities.

## Supplementary Materials

The following supporting information can be downloaded at: https://www.mdpi.com/article/10.3390/cancers18091415/s1, Figure S1: (A) Disease-free survival of patients tumor-free after primary therapy; (B) Disease-free survival stratified by FIGO stage; Table S1: Univariable and multivariable Cox regression for 5-year disease-free survival (DFS); Table S2: Exploratory sensitivity analysis of 5-year disease-free survival (DFS) according to histology (leiomyosarcoma vs. non-leiomyosarcoma); Table S3: Sensitivity analysis: comparison of baseline and outcome characteristics between patients with known vs. missing tumour grade data (n = 155); Table S4: Exploratory multivariable Cox proportional hazards regression for 5-year overall survival including tumour grade (n = 95).

## Abbreviations

CI, confidence interval; CT, computed tomography; CTX, chemotherapy; ECOG, Eastern Cooperative Oncology Group performance status; ESS, endometrial stromal sarcoma; FIGO, Fédération Internationale de Gynécologie et d’Obstétrique; HR, hazard ratio; ICD-O, International Classification of Diseases for Oncology; IQR, interquartile range; LMS, leiomyosarcoma; NCDB, National Cancer Database; NOS, not otherwise specified; OS, overall survival; RFS, recurrence-free survival; RT, radiotherapy; SEER, Surveillance, Epidemiology, and End Results; SPSS, Statistical Package for the Social Sciences.
