# Supplementary material for: Mapping Metastatic Spread in Uterine Sarcoma: A Population-Based Analysis of First Metastatic Patterns and Outcomes
Source: Cancers (Basel). 2026 Apr 29;18(9):1415. doi: 10.3390/cancers18091415 (PMC13162885; doi:10.3390/cancers18091415)
Supplement: Supplementary file 1 [file cancers-18-01415-s001.zip › Supplementary Figure S1 (A-B). Disease-free survival (DFS) analyses.pdf]

Supplementary Figure S1. Disease-free survival (DFS) analyses

Figure S1A. Disease-free survival of patients tumor-free after primary therapy.

Kaplan–Meier curve depicting disease-free survival calculated from the tumor-free date among patients who achieved tumor-free status after primary treatment.

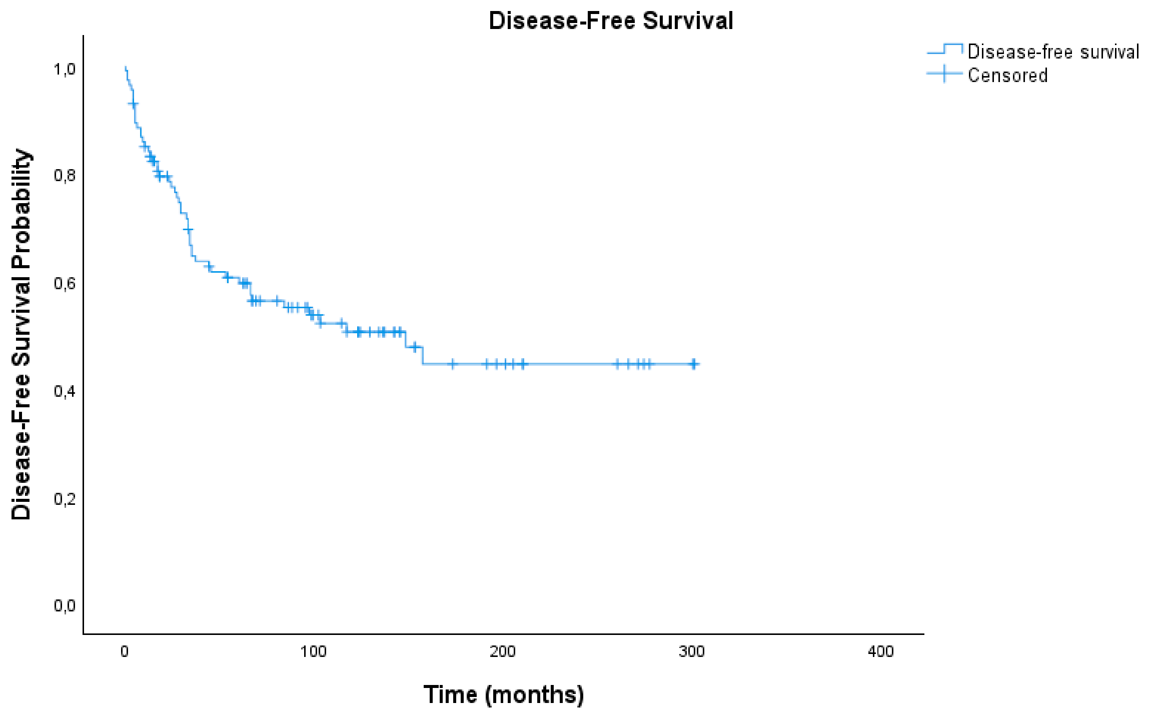

Figure S1B. Disease-free survival stratified by FIGO stage.

Kaplan–Meier curves comparing disease-free survival according to FIGO stage (I–II vs. III–IV) among patients who achieved tumor-free status after primary therapy.

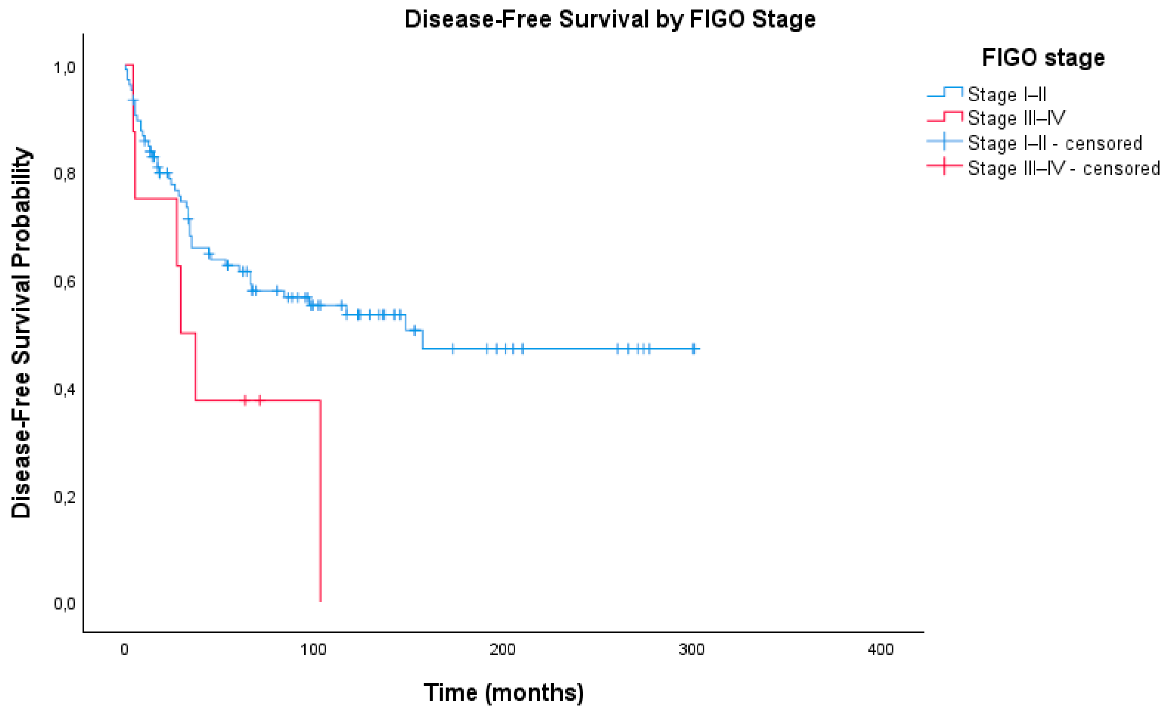

Log-rank p= 0.065
