# Supplementary material for: Mapping Metastatic Spread in Uterine Sarcoma: A Population-Based Analysis of First Metastatic Patterns and Outcomes
Source: Cancers (Basel). 2026 Apr 29;18(9):1415. doi: 10.3390/cancers18091415 (PMC13162885; doi:10.3390/cancers18091415)
Supplement: Supplementary file 1 [file cancers-18-01415-s001.zip › Supplementary Table S1. Univariable and multivariable Cox regression for 5-year disease-free survival (DFS).pdf]

Supplementary Table S1. Univariable and multivariable Cox regression for 5-year disease-free survival (DFS)

Variable	Univariable HR (95% CI)	p-value	Multivariable aHR (95% CI)	p-value
FIGO stage (III–IV vs I–II)	1.92 (0.76–4.89)	0.170	1.96 (0.77–4.99)	0.161
Histological group (HIST_GRP)	0.95 (0.67–1.35)	0.785	0.93 (0.65–1.34)	0.711
