# Supplementary material for: Mapping Metastatic Spread in Uterine Sarcoma: A Population-Based Analysis of First Metastatic Patterns and Outcomes
Source: Cancers (Basel). 2026 Apr 29;18(9):1415. doi: 10.3390/cancers18091415 (PMC13162885; doi:10.3390/cancers18091415)
Supplement: Supplementary file 1 [file cancers-18-01415-s001.zip › Supplementary Table S2. Exploratory sensitivity analysis of 5-year disease-free survival (DFS) according to histology (leiomyosarcoma vs non-leiomyosarcoma).pdf]

Supplementary Table S2. Exploratory sensitivity analysis of 5-year disease-free survival (DFS) according to histology (leiomyosarcoma vs non-leiomyosarcoma)

Variable	Univariable HR (95% CI)	p value	Multivariable aHR (95% CI)	p value
Histology (leiomyosarcoma vs non-leiomyosarcoma)	1.74 (0.96–3.18)	0.069	1.94 (1.04–3.61)	0.036
FIGO stage (III–IV vs I–II)	1.92 (0.76–4.89)	0.170	2.45 (0.93–6.45)	0.069

This exploratory sensitivity analysis was restricted to patients who achieved tumor-free status after primary therapy (n = 114; 43 DFS events). Follow-up was administratively censored at 60 months. Leiomyosarcoma was compared with all other uterine sarcoma histologies. Multivariable models were adjusted for FIGO stage only due to the limited number of events. Results should be interpreted cautiously given the exploratory and post-hoc nature of the analysis.
