# Supplementary material for: Mapping Metastatic Spread in Uterine Sarcoma: A Population-Based Analysis of First Metastatic Patterns and Outcomes
Source: Cancers (Basel). 2026 Apr 29;18(9):1415. doi: 10.3390/cancers18091415 (PMC13162885; doi:10.3390/cancers18091415)
Supplement: Supplementary file 1 [file cancers-18-01415-s001.zip › Supplementary Table S3. Sensitivity analysis.pdf]

Supplementary Table S3. Sensitivity analysis: comparison of baseline and outcome characteristics between patients with known vs. missing tumour grade data (n=155).

Variable	Grade known (n=97)	Grade missing (n=58)	p value	Test
**Histologic subtype**				
Leiomyosarcoma	38 (39.2%)	29 (50.0%)	**0.005***	χ^2
Endometrial stromal sarcoma	44 (45.4%)	13 (22.4%)		
Adenosarcoma	5 (5.2%)	11 (19.0%)		
Other/undifferentiated	10 (10.3%)	5 (8.6%)		
FIGO stage III–IV	25 (25.8%)	18 (31.0%)	0.479	χ^2
Metastatic disease	32 (33.0%)	22 (37.9%)	0.532	χ^2
Deceased	45 (46.4%)	32 (55.2%)	0.290	χ^2
Age at diagnosis, years – median (IQR)	59 (51–70)	60 (50–73)	0.631	M-W U

* Statistically significant ($p < 0.05$). Values are n (%) unless otherwise stated. χ^2 = Pearson chi-square test. M-W U = Mann–Whitney U test. IQR = interquartile range. Missing tumour grade was significantly associated with histologic subtype only (p=0.005), with adenosarcoma showing the highest proportion of missing data (68.8%), consistent with the absence of a standardised grading system for this subtype. No significant differences were observed for FIGO stage, metastatic status, overall survival, or age at diagnosis, supporting the assumption that grade-based analyses are not substantially biased by the missing data pattern for the primary prognostic endpoints.
