# Supplementary material for: Mapping Metastatic Spread in Uterine Sarcoma: A Population-Based Analysis of First Metastatic Patterns and Outcomes
Source: Cancers (Basel). 2026 Apr 29;18(9):1415. doi: 10.3390/cancers18091415 (PMC13162885; doi:10.3390/cancers18091415)
Supplement: Supplementary file 1 [file cancers-18-01415-s001.zip › Supplementary Table S4. Exploratory multivariable Cox proportional hazards regression for 5-year overall survival including tumour grade.pdf]

Supplementary Table S4. Exploratory multivariable Cox proportional hazards regression for 5-year overall survival including tumour grade (n=95).

Variable	HR	95% CI	p value
FIGO stage (III–IV vs I–II)	**4.15**	1.79–9.62	**0.001***
Tumour-free after primary therapy (yes vs no)	0.67	0.29–1.53	0.344
Tumour grade (high vs low)	**4.84**	1.85–12.63	**0.001***

* Statistically significant ($p < 0.05$). HR = hazard ratio. CI = confidence interval. This exploratory analysis was restricted to 95 patients with complete data for all three covariates (60 patients excluded due to missing tumour grade). Administrative censoring at 60 months. Events: 38 deaths in 95 patients (events-per-variable ratio ≈ 13). Tumour-free status after primary therapy did not retain independent prognostic significance in this reduced model (p=0.344), likely reflecting the reduced sample size and the correlation between tumour grade and the probability of achieving tumour-free status. Results should be interpreted cautiously given the exploratory and post-hoc nature of the analysis.
